# Reattachment of a Vertical Complicated Subgingival Crown Root Fracture in a 10-Year Old Child: A Case Report

**DOI:** 10.5005/jp-journals-10005-1020

**Published:** 2009-12-26

**Authors:** Vishwas Chaugule, Chetan Bhat, Vishwas Patil, Sajjad H Mithiborwala

**Affiliations:** 1 Professor, Head and PG Guide, Department of Pedodontics and Preventive Dentistry, Dr DY Patil Dental College and Hospital Pimpri, Pune-411018, Maharashtra, India; 2 Lecturer, Department of Pedodontics and Preventive Dentistry, Dr DY Patil Dental College and Hospital, Pimpri Pune-411018, Maharashtra, India; 3 Lecturer, Department of Pedodontics and Preventive Dentistry, Dr DY Patil Dental College and Hospital, Pimpri Pune-411018, Maharashtra, India; 4 Lecturer, Department of Pedodontics and Preventive Dentistry, Terna Dental College and Hospital, Nerul, Navi Mumbai Maharashtra, India

**Keywords:** Biologic restoration, complicated subgingival, crown-root fracture, fragment reattachment.

## Abstract

Functional, esthetic and biologic restoration of a fractured incisor often presents a daunting clinical challenge. The outcome of conventional composites, prosthodontic restorations in a young patients result in an uncertain longevity of the same. Reattachment of the fractured fragment of a tooth helps in maintaining both morphology and esthetics in a growing child until the permanent long lasting solution is sought after the complete development of the dentition and the jaws. Since fractured fragment exhibited no caries, not even negligible loss of tooth structure and was adapting well to the remaining tooth structure when tried in, the reattachment of fractured fragment was considered as a viable treatment option. This treatment option for complicated subgingival crown-root fracture depicts the involvement of periodontal surgical exposure, endodontic management and ultimately the fragment reattachment.

## INTRODUCTION

A crown-root fracture is defined as a fracture involving enamel dentin and cementum and is classified as complicated or uncomplicated, according to the pulpal involvement and comprises 5% of the traumatic injuries affecting the permanent dentition and 2% in the primary dentition.^1^

Trauma to oral structures like teeth poses a great psychological impact on the minds of patients, more so particularly when they are children. Since anterior teeth help in maintaining the form, function and esthetics, a treatment plan which would not compromise on any of these values will turn out to be a desirable one.

A few approaches have already been established for such types of fractures like orthodontic extrusion, forced surgical extrusion and periodontal crown lengthening procedures to expose the fracture site followed by restoring the lost tooth structure by prefabricated or custom cast post and core build up with, composite resin or prosthodontic restoration but each is not without inherent drawbacks like, excessive forces in orthodontic extrusion can lead to pain, failure of the tooth to move, root damage, tilting of the abutment and subsequent impaction of the root being extruded. The custom or prefabricated post and core have hazards like Possibility of root perforation during the post space preparation, induced stresses and the risk of fracture during the placement of the post and wedging effects of the tapered posts.^2^,^3^ Also these approaches turn out to be time consuming, elaborate and not so very cost effective.

Prosthodontic restoration in such cases involving younger patient is questionable because of larger pulpal sizes, progressive eruption and gingival margin instability.^4^

The era of composite resins have opened a lot of vistas for different treatment approaches.

When an intact fragment is available its attachment may offer a most functional and esthetic treatment option.^4^

The present narrated approach happens to be one of the favorable applications of the adhesive restorative dentistry.

## CASE REPORT

A 10 years old healthy female patient with no contributory medical history visited the Department of Pediatric and Preventive Dentistry, Dr DY Patil Dental College Pune, with the chief complaint of injury to the upper front tooth 15 days back and discomfort while chewing.

**Fig. 1:**
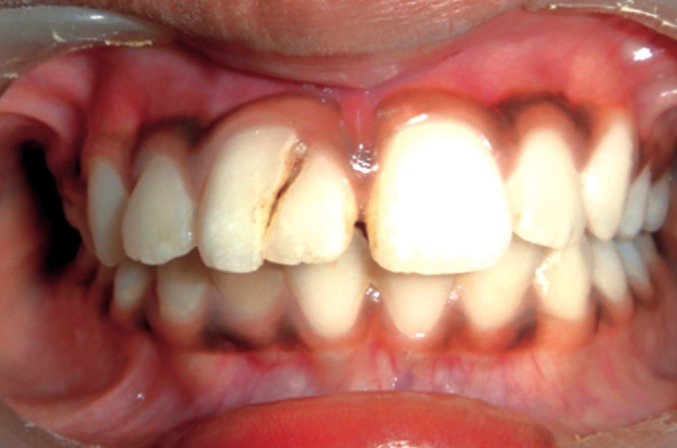
Labial view of the fractured tooth

**Fig. 2:**
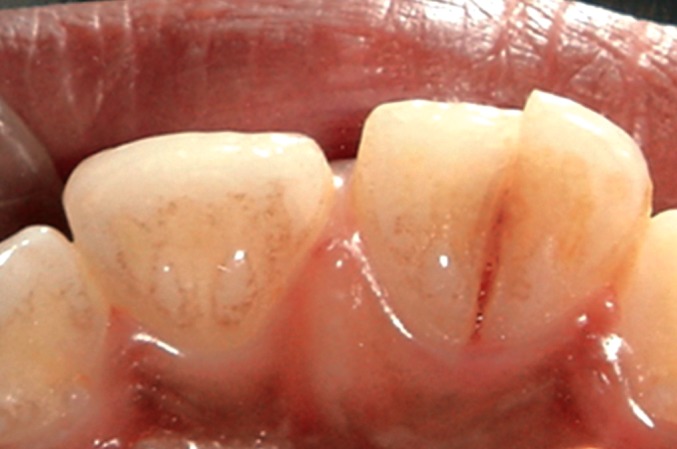
Palatal view of the fractured tooth

On examination, the upper right central incisor showed a complicated crown-root fracture extending from the center of incisal edge going up to and beyond the cervical line subgingivally (Figs1 and 2). The fractured fragment showed grade 3 mobility while the remaining part of tooth structure exhibited grade 2 mobility.

**Fig. 3:**
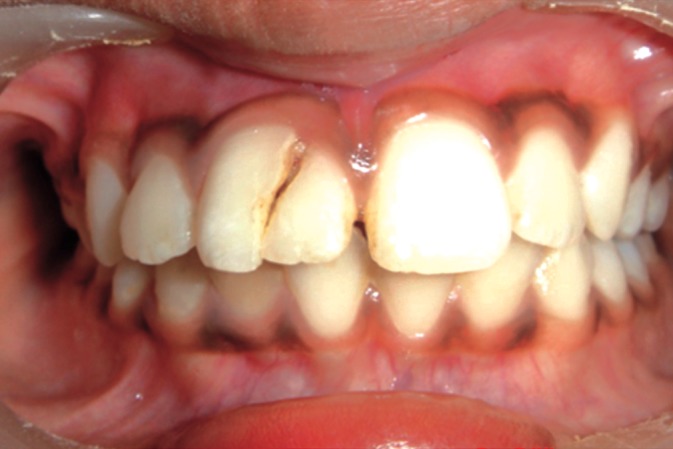
Occlusion

The involved tooth was tender on vertical percussion however occlusion was unaffected (Fig. 3).

**Fig. 4:**
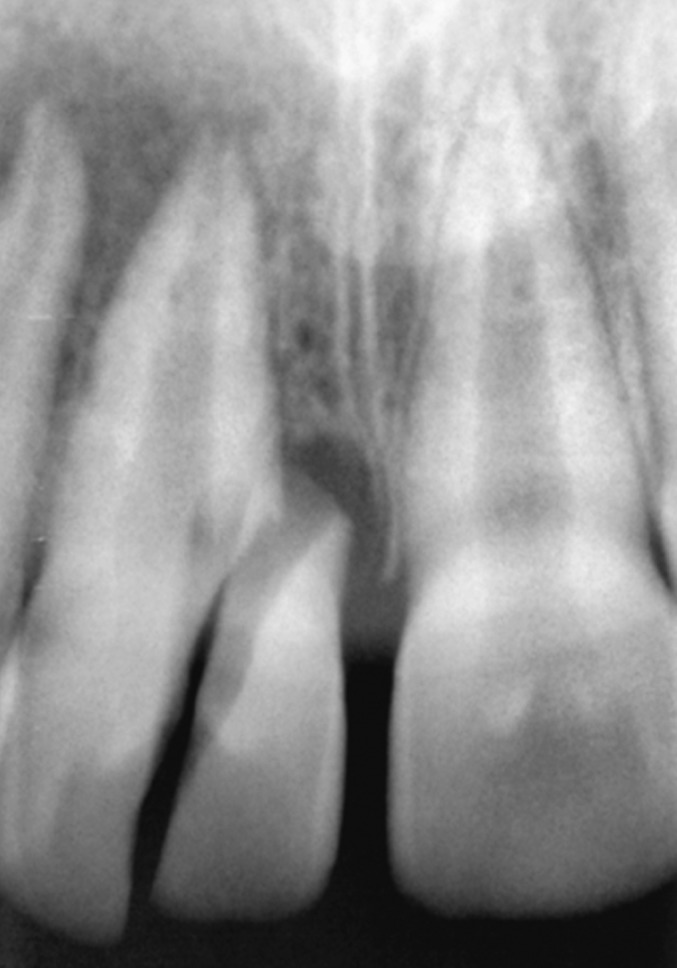
Preoperative radiograph of the fractured tooth

Radiographic examination of the upper anterior region revealed the fracture line in upper right central incisor passing from its incisal edge, through the pulp and extending into the cervical one third of the root. Periapical area showed a marginal rarefaction suggestive of inflammatory change or slight extrusion of the tooth (Fig. 4).

Routine hematological investigations (Hemoglobin, total RBC count, total and differential WBC count, clotting time, bleeding time) showed all the values within the normal range.

Various treatment modalities were explained to the parents of the child with their pros and cons and it was finally decided to go ahead with the present treatment approach.

Preparation of the operation site was done by scrubbing with 2% povidone-Iodine solution and profound anesthesia was achieved by administering 2% Lignocaine with Adrenaline (1:200000).

**Fig. 5:**
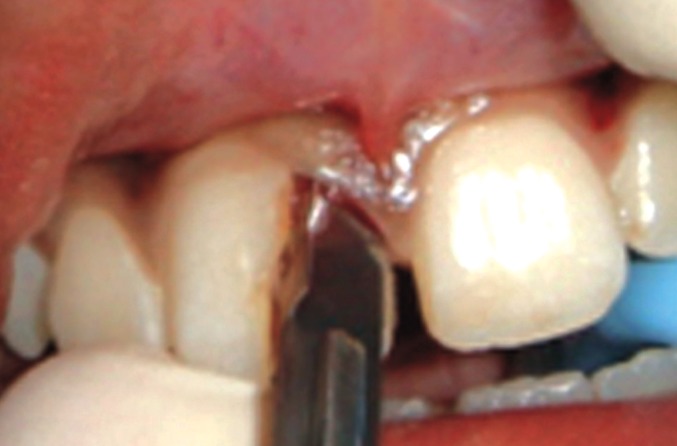
Sulcular incision

The fractured fragment was detached from the main tooth structure by separating the gingival attachment with no. 15 BP blade (Fig. 5).

The fractured fragment was then cleaned of debris and kept soaked in normal saline. It measured about 3-4 mm below cementoenamel junction.

**Fig. 6:**
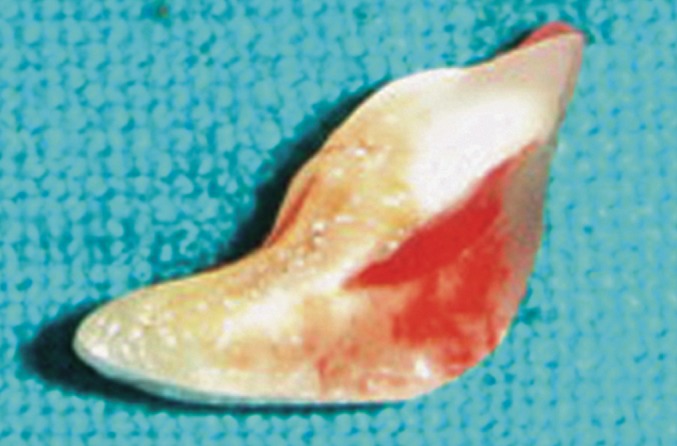
Removed fragment

**Fig. 7:**
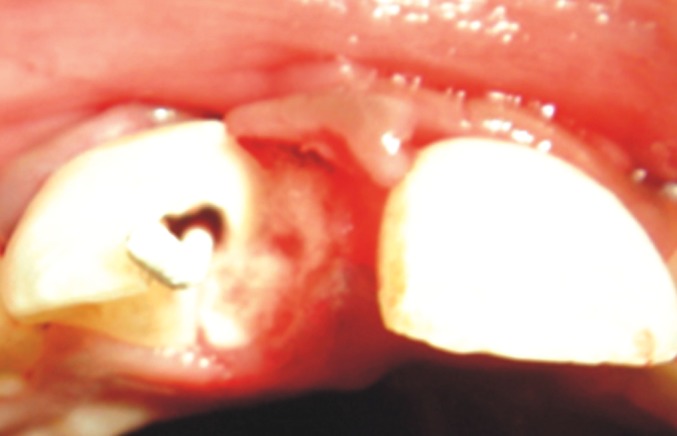
Subgingival curettage of the fracture area

**Fig. 8:**
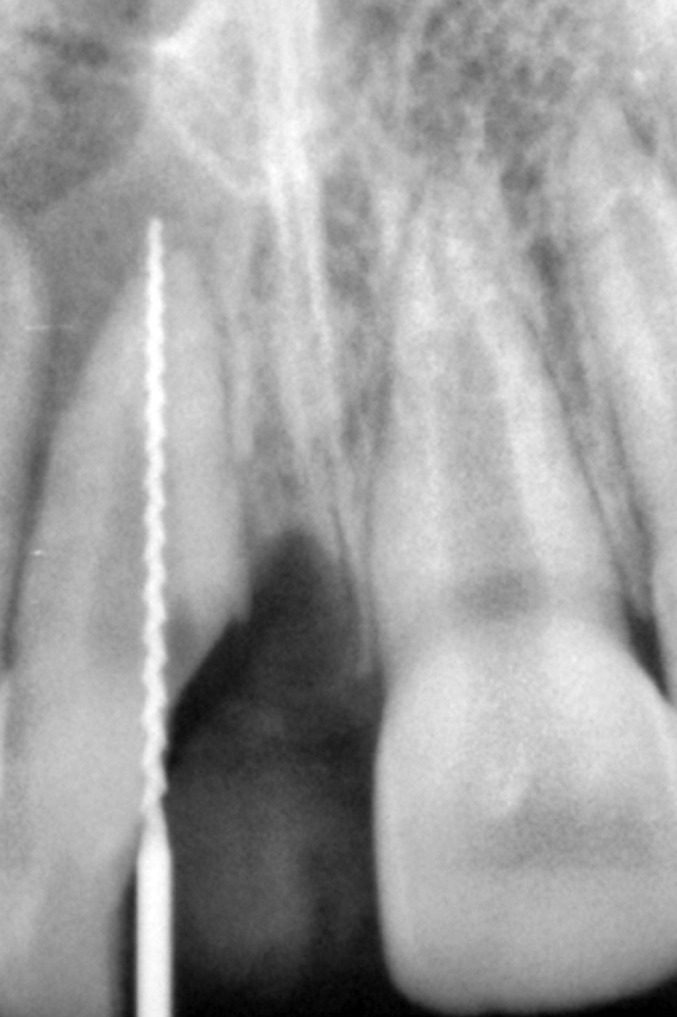
Endodontic treatment of the involved tooth

**Fig. 9:**
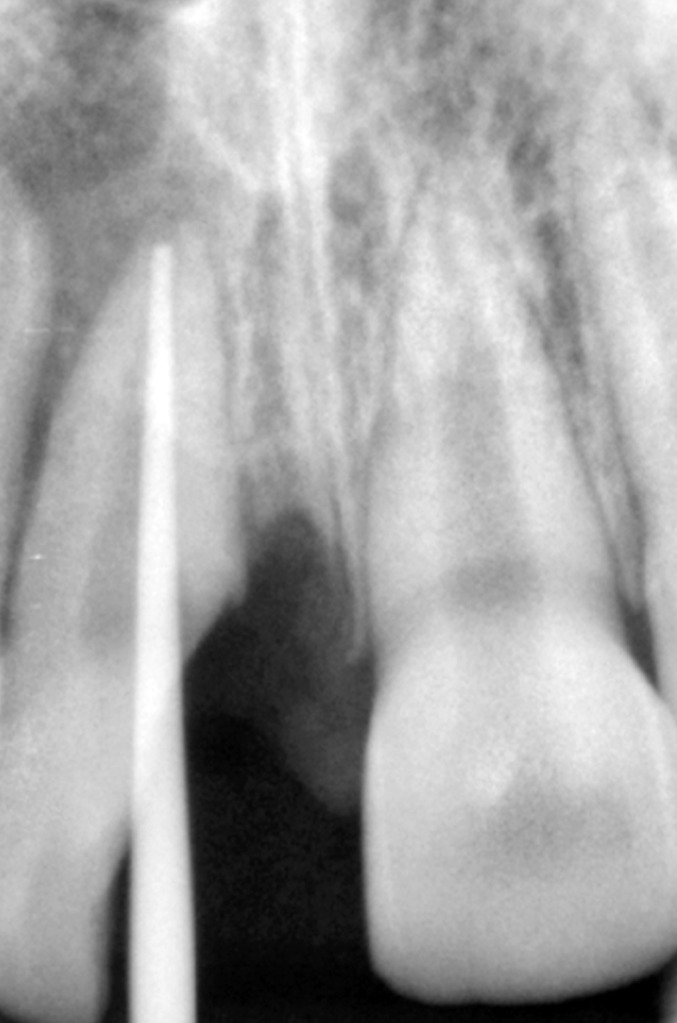
Endodontic treatment of the involved tooth

The sulcular incision was extended and single papilla flap was raised on labial and palatal aspect to expose the fracture margin (Fig. 6).

Subgingival curettage was done to remove the encroached soft tissue in the fractured segment near the alveolar crest on the medial aspect of the involved central incisor (Fig. 7).

The fractured area was then contoured with a round SS bur no. 6 to restore the lost biologic width, since the tooth contouring minimizes the removal of the supporting alveolar bone.^5^,^6^

Hemorrhage was then controlled using ferric sulphate (Astringedent, Ultradent, USA).

Endodontic treatment of the fractured tooth was performed as for the single visit protocol (Figs 8 and 9).

Calcium hydroxide and iodoform paste (Vitapex, J Morita, Japan) was used in apical one third and little area beyond that as the periapical area showed apical rarefaction.

AH-26 (Dentsply, USA) was used as a sealer and the canal was obturated using gutta-percha points. The canal orifice was sealed with glass ionomer cement (GC Corp Tokyo, Japan).

The subgingival extensions of the main tooth structure and the fractured fragment were left untouched for the purpose of a guide to the reattachment.

**Fig. 10:**
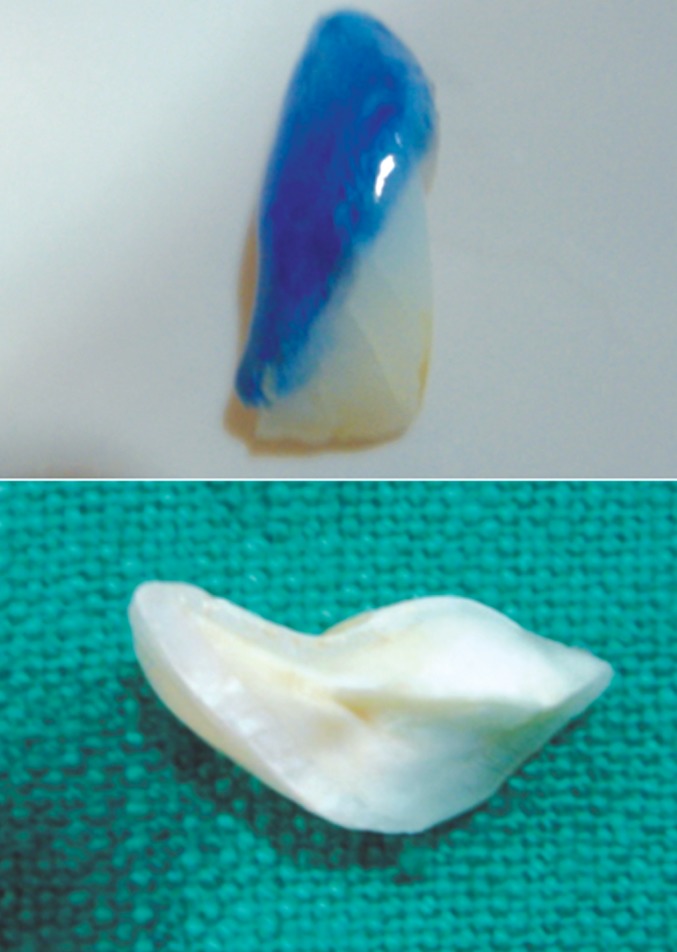
Application of etchant on fractured fragment

The fractured fragment and the main tooth were cleaned using pumice and water slurry.^4^ Surfaces of the fractured fragment and the main tooth structure were etched with 34% phosphoric acid gel (Dentsply, USA) for 15 seconds, washed and dried moist (Fig. 10).

Later they were coated with Prime and Bond NT (Dentsply, USA) with the help of applicator tip and cured for 20 seconds.

**Fig. 11:**
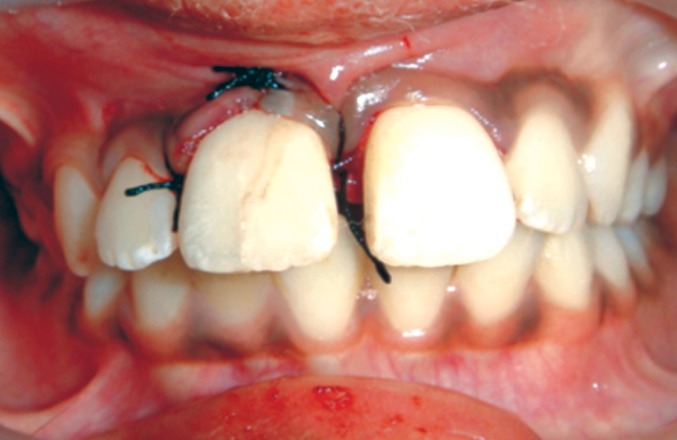
Fragment reattachment and repositioning of flap

The fractured fragment was bonded to the remaining tooth structure with the help of the microfilled anterior composite (Clearfill, Kuraray, Japan) which was photo polymerized as per the instructions of the manufacturer (Fig. 11).

The flap was sutured back with 3-0 black silk.

**Fig. 12:**
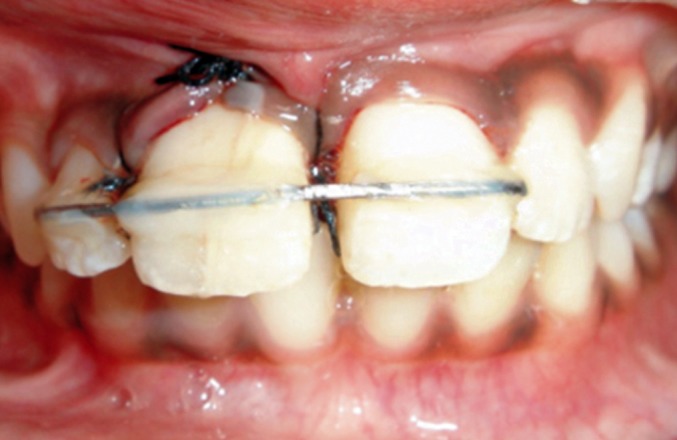
Splinting of the involved tooth

**Fig. 13:**
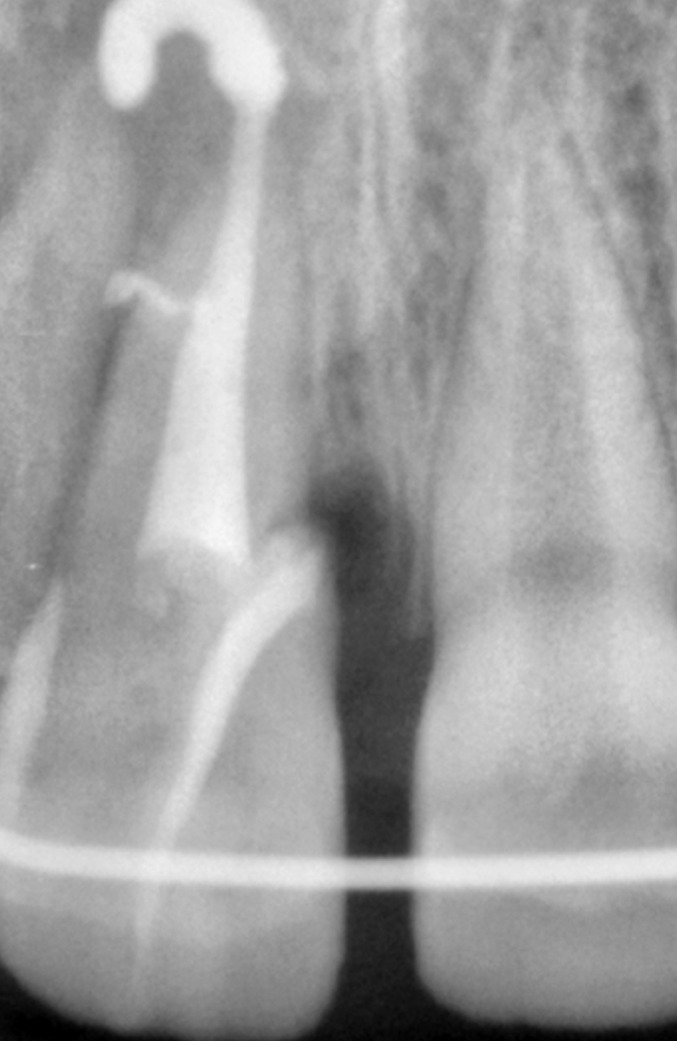
Postoperative radiograph of the involved tooth

Since the involved tooth exhibited grade 2 mobility it was splinted to the adjacent lateral and central incisor with the help of 21 gauge SS wire and composite resin (Figs 12 and 13).^7^,^8^

Patient was advised to maintain oral hygiene by using 0.2% chlorhexidine gluconate mouthwash twice daily after gentle brushing after every meal. Patient was also instructed to avoid sticky and hard food substances until the removal of the splint.

**Fig. 14:**
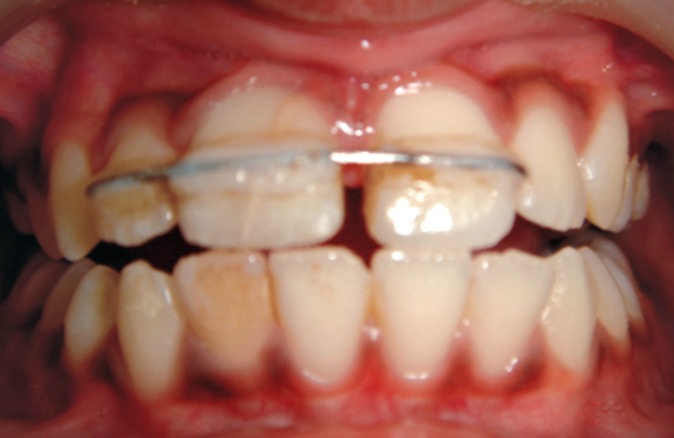
Labial view of the involved tooth after suture removal

**Fig. 15:**
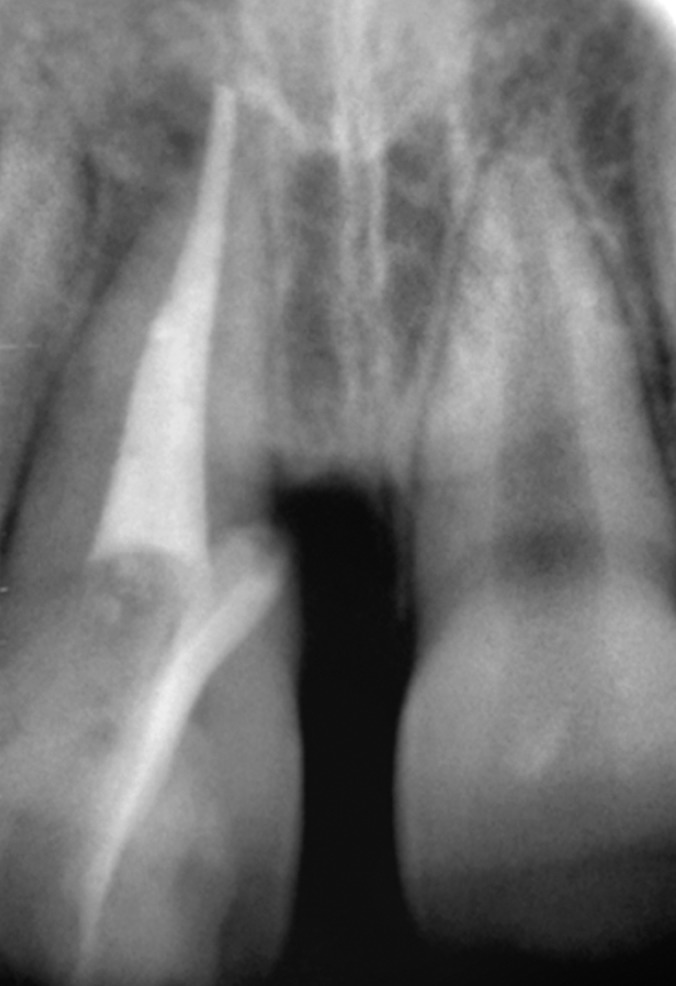
Radiograph of the tooth after splint removal

Initially patient was called after 7 days for suture removal and two weeks thereafter for the removal of the splint (Figs 14 and 15).

**Fig. 16:**
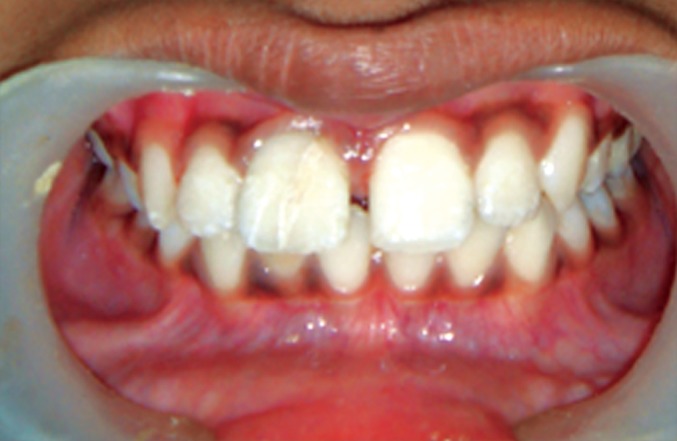
Labial view of the involved tooth 3 weeks postoperatively

After the splint removal demarcating line between fractured segment and unaffected part of tooth became apparent (Fig.16).

**Fig. 17:**
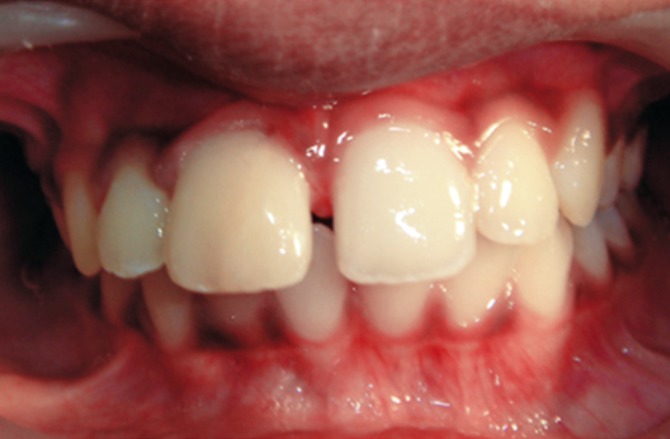
Labial view after masking the demarcating line 3 weeks postoperatively

Thereafter it was masked with additional layer of the composite (Clearfill, Kuraray, Japan) which resulted into a fairly acceptable aesthetics (Fig. 17).

## DISCUSSION

Various studies regarding the incidence of dental trauma, especially in the pediatric and adolescent populations, have made it clear that this injury is of significant nature and effects up to one third of patients in this age group. Studies have reported estimates that about one out of every four persons under age of 18 will sustain a traumatic dental injury in the form of an anterior crown fracture.^9^-^11^

The most common etiological factors of crown-root fractures are falls, automobile and bicycle accidents.

In anterior teeth crown-root fracture is caused by direct trauma while in posterior teeth it is caused by indirect trauma.

Fracture lines seen in crown-root fractures can be single or multiple and commonly seen in horizontal direction.

A rare type of injury is a vertical fracture of crown-root running along the long axis of the tooth or deviating in a mesial or distal aspect.^1^

The traditional approach in restoring a crown-root fracture is by using cast post or prefabricated post and a core buildup but this procedure has numerous disadvantages as mentioned earlier.

Andreasen and Andreasen stated that reattachment of fractured segment serves as a transitional treatment alternative for preteens or teenage patients to postpone definitive treatment until an age where gingival margin contours are relatively stable.^12^

Excessive forces if used during orthodontic extrusion of roots can lead to pain, failure of the tooth to move, root damage, tilting of the abutment teeth and subsequent impaction of the root being extruded so this treatment option was ruled out by the parents.^13^

As the reattachment procedure does not preclude any future treatment so whenever an intact fragment is available, reattachment of fractured fragment should be considered as a viable first treatment option.^4^

Liew too described the prognosis of this procedure as it can act as ‘a short to medium term temporary restoration which has the potential for indefinite service'.^14^

Therefore the narrated approach was opted.

**Fig. 18:**
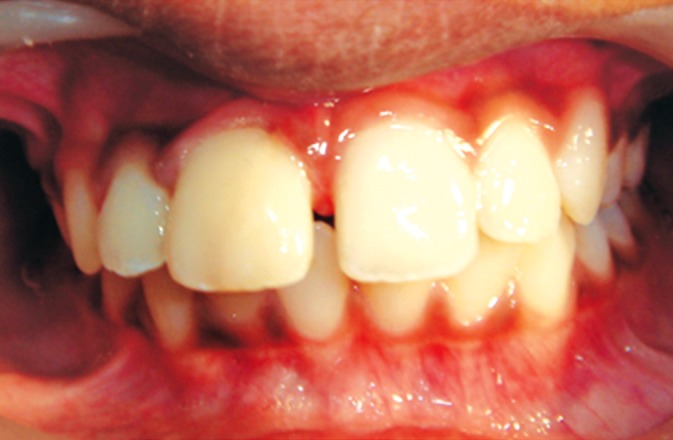
Labial view of the involved tooth 3 months postoperatively

Although the application of rubber dam for the purpose of isolation provides an environment conducive to qualify adhesive dentistry. In this case it was not to be used because the base of the fracture line was too subgingival and the application of rubber dam clamp would have lead to excessive uncontrolled bleeding from the soft tissues hence other means of isolation such as cotton rolls, 2 x 2 gauze and high vacuum suction were used along with the hemostatic agent.

**Fig. 19:**
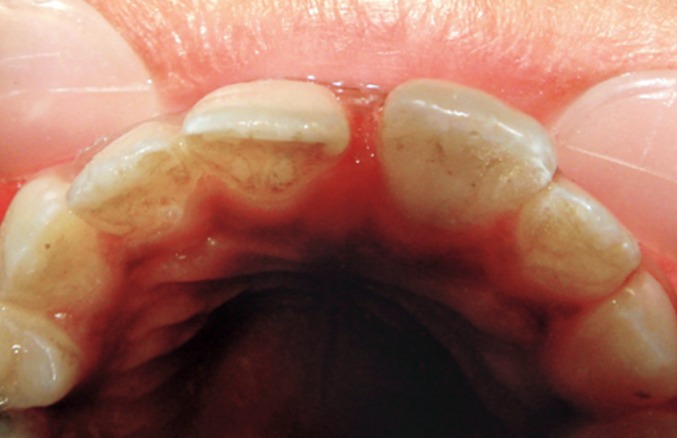
Palatal view of the involved tooth 3 months postoperatively

**Fig. 20:**
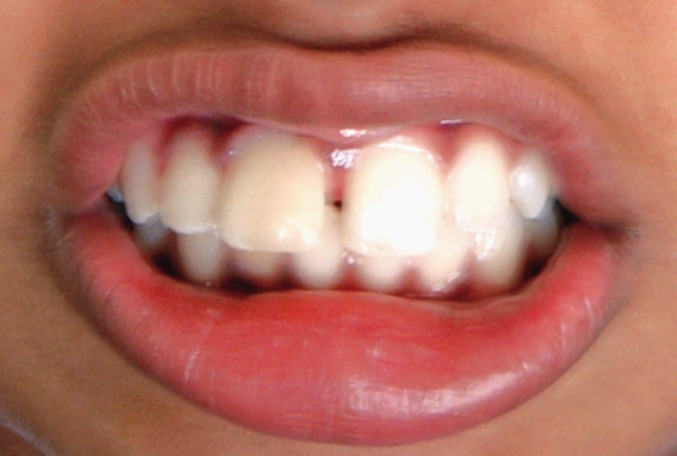
Front view of the patient 6 months postoperatively

It remains to be seen the extent of the longevity of the reattachment prior to the final permanent restoration in the form of a suitable extracoronal restoration.

## CONCLUSION

Although the reattachment is susceptible to the effects of cyclic fatigue and hydrolytic degradation over time, various studies have described functional and esthetic successes exceeding 7 years.^4^ In the present case "Patient came for the follow-up at the end of the third month (Figs 18 and 19) and 6 months (Fig. 20)" respectively. The prognosis of reattachment procedure further needs to be assessed in longterm clinical studies.

**What this paper adds**

The fragment reattachment procedure offers the advantages like cost effectiveness, a viable option for maintenance of esthetics and the conservation of natural tooth structure. For general dentist the treatment modality described could be considered as one of the interim one until the final prosthesis is given without sacrificing too much of time.

**Why this paper is important for pediatric dentists**

Trauma to oral structures like teeth poses a great psychological impact on the minds of patients, more so particularly when they are children. Therefore it is the responsibility of a pediatric dentist to opt for such a treatment modality which will be less traumatic in an already existing traumatic situation, cost effective, easy to perform and ultimately resulting into an esthetically acceptable outcome. This paper provides one of such options.
